# Braided stent-assisted coil embolization versus laser engraved stent-assisted coil embolization in patients with unruptured complex intracranial aneurysms

**DOI:** 10.1016/j.clinsp.2023.100202

**Published:** 2023-04-30

**Authors:** Jie Zhang, Ling He, Xun Xia, Lie Zhang, Kai Yu

**Affiliations:** a The First Affiliated Hospital of Chengdu Medical College, Department of Neurosurgery, Chengdu, Sichuan, China; b The First Affiliated Hospital of Chengdu Medical College, Department of Hematology, Chengdu, Sichuan, China

**Keywords:** Braided stents, Endovascular therapy, Intracranial aneurysms, Laser-cut stents, Magnetic resonance angiography, Stent-assisted coil embolization, Subarachnoid hemorrhage

## Abstract

**Purposes:**

Braided and laser-cut stents both are efficacious and safe for coiling intracranial aneurysms. The study aimed to compare outcomes following braided stent-assisted coil embolization *versus* laser engraved stent-assisted coil embolization in 266 patients who were diagnosed with unruptured intracranial aneurysms of different types and locations.

**Methods:**

Patients with unruptured complex intracranial aneurysms underwent braided (BSE cohort, *n* = 125) or laser engraved (LSE cohort, *n* = 141) stent-assisted embolization.

**Results:**

The deployment success rate was higher for patients of the LSE cohort than those of the BSE cohort (140 [99%] vs. 117 [94%], *p* = 0.0142). Seventy-one (fifty-seven percentages) and 73 (52%) were coil embolization procedure success rates of the BSE and the LSE cohorts. Periprocedural intracranial hemorrhage was higher in patients of the BSE cohort than those of the LSE cohort (8 [6%] vs. 1 [1%], *p* = 0.0142). Four (three percentages) patients from the LSE cohort and 3 (2%) patients from the BSE cohort had in-stent thrombosis during embolization. Permanent morbidities were higher in patients of the LSE cohort than those of the BSE cohort (8 [6%] vs. 1 [1%], *p* = 0.0389). Higher successful procedures (76% vs. 68%) and fewer postprocedural intracranial hemorrhage (0% vs. 5%) and mortality (0% vs. 5%) were reported for patients of the BSE cohort in posterior circulation aneurysmal location than those of the LSE cohort. Laser engraved stent has fewer problems with deployment and may have better periprocedural and follow-up outcomes after embolization.

**Conclusions:**

Braided stent-assisted embolization should be preferred when the aneurysm is present in the posterior circulation.

## Highlights

• Laser engraved stent has easy deployment & has better follow-up outcomes.
• Success of coil embolism would be higher for braided stent-assisted embolization.
• Braided stent should be preferred for the posterior circulation aneurysm.

## Introduction

Complex intracranial aneurysms and rupture of aneurysms lead to subarachnoid hemorrhages and have serious effects on the quality of life of patients.^1^, ^2^, ^3^ Coil embolization is endovascular therapy generally used for intracranial aneurysms.^4^ Complex intracranial aneurysms are giants in size and are difficult to manage by coil embolization.^5^ There are so many complications during embolization, for example, aneurysm rupture, subarachnoid hemorrhage, and thrombosis^6^ Several endovascular devices are used during endovascular therapies^4^ Current endovascular therapies include endovascular coil embolization and craniotomy^7^ Although the advances in craniotomy procedures, endovascular coil embolization is preferred for intracranial aneurysms because of the minimally invasive procedure,^8^ good patient acceptance of the technique, and better clinical outcomes^9^ Open-cell type, closed-cell type, laser-cut type, braided type, pore size type, metal coverage type, etc. intracranial stents have been used by neurosurgeons for mechanical support during endovascular coil embolization.^10^ The main functions of intracranial stents are preventing the collapse of a coil, divert of blood flow around aneurysms, and scaffolding for endothelial growth.^11^ Although several advancements in stent technology, stent expansion and stenosis are major issues with stent-assisted coil embolization.^12^ Braided stents and laser-cut stents both have been reported as efficacious and safe for stent-assisted coiling of intracranial aneurysms.^13^

Braided stents have lower events of permanent morbidity.^13^ For small-sized intracranial aneurysms (< 10 mm) among Korean patients, braided stents with closed cells have favorable postoperative outcomes as compared to open-cell or laser-cut stents.^14^ Also, laser-cut stents have a higher rate of successful deployment and lower events of periprocedural intracranial hemorrhage.^13^ Efficacy and safety analysis of braided stent and laser engraved stent in coil embolization among Chinese patients with complex intracranial aneurysms are not been adequately investigated yet^10^,^15^ and there are still controversies regarding the effects of the braided stent and laser engraved stent in coil embolization because both are closed type stents.^10^

The objectives of the current retrospective study were to compare the demographical, clinical, and angiographic characteristics of patients before endovascular coil embolization, deployment success rate, periprocedural complications, follow-up outcomes, and recanalization rate of patients with unruptured complex intracranial aneurysms who underwent braided stent-assisted embolization against those of patients who underwent laser engraved stent-assisted embolization. Also, to analyze procedural success rate and follow-up outcomes according to aneurysmal location.

## Materials and methods

### Ethics approval and consent to participate

The designed protocol of the current study was approved by the Chengdu Medical College review board (Approval n° BMC15148 dated 15 January 2018). The study design follows the law of China, the V2008 Declarations of Helsinki. In a retrospective analysis, there is no need for the requirement of a consent form from patients.

### Inclusion criteria

Patients with complex intracranial aneurysms who underwent stent-assisted embolization (braided or laser-engraved stent) were included in the analysis.

### Exclusion criteria

Patients with incomplete details were excluded from the study. Patients with complex but non-saccular aneurysms were excluded from the analysis. Patients who had required re-treatment for endovascular therapy were excluded from the study.

### Characteristics of patients

Demographical parameters, clinical characteristics, patients’ behavior, and angiographic parameters before endovascular coil embolization were collected and analyzed.

### Endovascular coil embolization

General anesthesia (propofol-based anesthesia) was induced in all patients. Arterial architectural details and configurations of complex aneurysms were evaluated using cerebral and rotational angiography using three-dimensional image reconstruction. Maximal dimensions of complex aneurysms were found using three-dimensional angiographic depictions. Digital subtraction angiography was used to access depth and neck sizes. Intra-procedural 3000 IU of intravenous heparin was administered. A dual antiplatelet agent was prescribed post-surgery for 3 months and the single antiplatelet agent was prescribed post-surgery for 1 year.

Braided stents and laser-cut stents both were nickel and purchased from Peiertech, Jiangsu, China. For all LSE cases and for BSE cases the stents used were the same. The deployment procedure for the stents was the same.

### Follow-up study

#### Occlusion

The Raymond classification was used to evaluate initial angiographic occlusive results after the procedure. The Raymond classification is graded as complete occlusion, residual aneurysm, or residual neck.^16^ The Raymond classification grading of complete occlusion and residual neck were considered a successful procedure. Magnetic resonance angiography was performed at 6 months, 12 months, 18 months, 24 months, and 36 months after the procedure. If magnetic resonance angiography reported suspected results digital subtraction angiography was performed as confirmatory imaging. Then after the decision to retreatment was made. The reading of radiologists was assessed for retreatment.

#### Adverse effects

Procedural-related and diseases related adverse effects were evaluated from the medical records of the patients.

#### Recanalization

The numbers of patients who underwent recanalization were evaluated from the medical records of the patients.

### Statistical analyses

InStat 3.01, GraphPad Software, San Diego, CA, USA was used for statistical analysis purposes. Categorial variables were analyzed by the Chi-Square test (*χ*^2^-test) or Fisher's exact test. Gaussian distributions were tested using Kolmogorov and Smirnov methods. For linear continuous data with equal Standard Deviations (SDs) unpaired *t*-test was performed. For linear continuous data with unequal SDs, the unpaired *t*-test with Welch correction was performed. For not linear continuous data Mann-Whitney test was performed. All results were considered significant if the p-value was less than 0.05.

## Results

### Study population

**Fig. 1:**
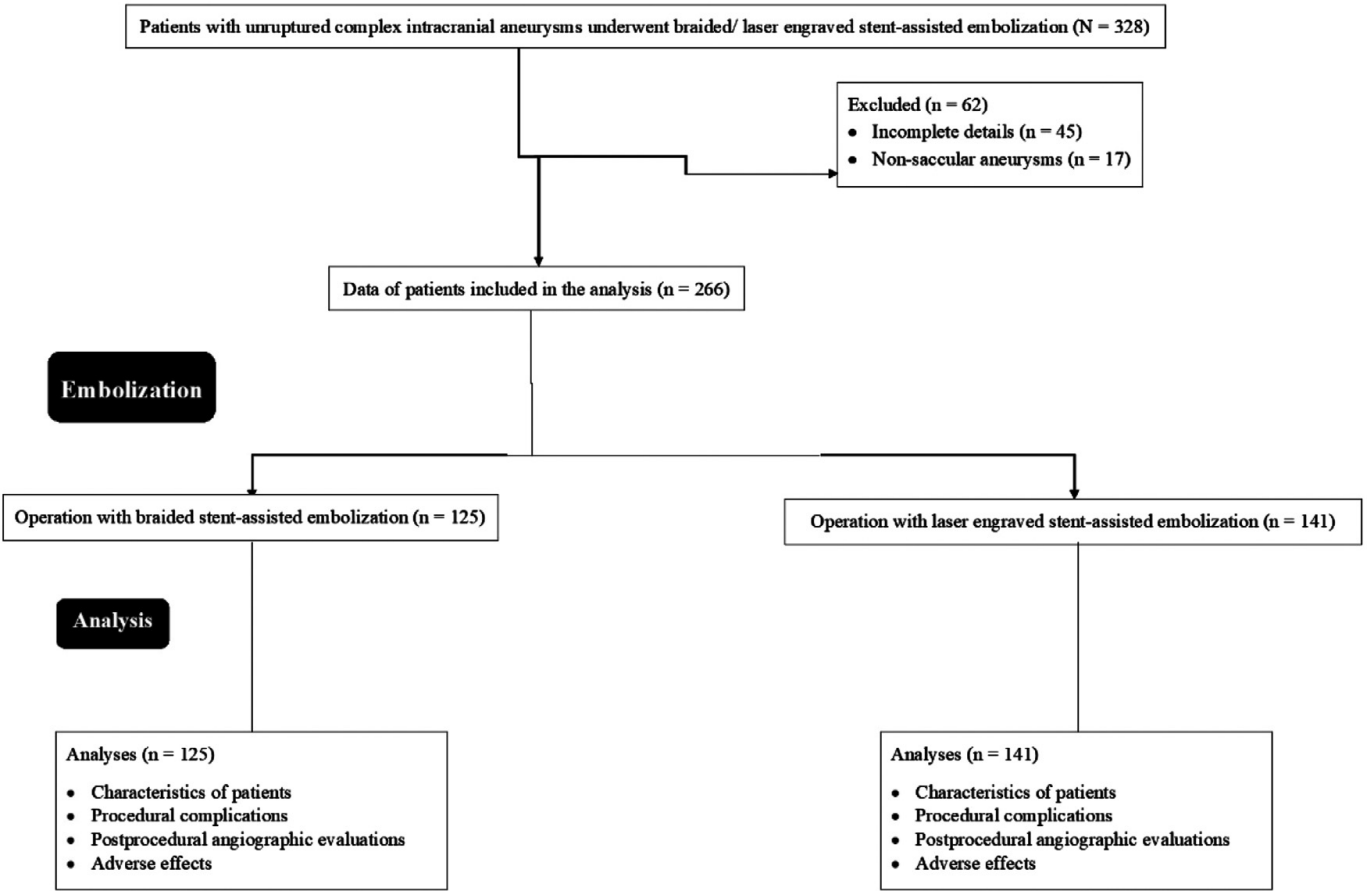
Study summary chart.

From 21 January 2018 to 5 October 2019, a total of 328 patients with unruptured complex intracranial aneurysms underwent braided or laser-engraved stent-assisted embolization at the First Affiliated Hospital of Chengdu Medical College, Chengdu, Sichuan, China, and the referring hospitals. Among 328 patients, 45 patients had incomplete details in hospital records, and 17 patients had non-saccular aneurysms. Therefore, the data from 62 patients were excluded from the study. Among 266 patients, a total of 125 patients underwent operation with braided stent-assisted embolization and a total of 141 patients underwent operation with laser engraved stent-assisted embolization. Characteristics of patients and follow-up study parameters were evaluated for 266 patients. The study summary chart is presented in Fig. 1.

### Characteristics of patients

A total of 155 (58%) males and 111 (42%) females underwent coil embolization. The median age of patients was 62 years (Q3: 67 years, Q1: 57 years). Hypertension was reported in 119 (45%) patients. A total of 22 (8%) and 80 (30%) patients were reported diabetic and hyperlipidemic. Only 19 (7%) patients were current smokers. Before embolization, demographical and clinical characteristics of patients and patients’ behavior have insignificant differences between patients who either underwent Braided Stent-assisted Embolization (BSE cohort) or underwent Laser Engraved Stent-assisted embolization (LSE cohort; *p* > 0.05 for all, Fisher's exact test, or χ2-test, or Mann-Whitney test, Table 1). A total of 226 (85%) aneurysms were in the anterior circulation and 40 (15%) aneurysms were in the posterior circulation. Median aneurysm size was 5.5 mm (Q3: 7 mm; Q1: 4 mm). Median neck size was 4 mm (Q3: 5 mm; Q1: 3 mm). Median depth-to-neck was 1.2 mm (Q3: 1.3 mm; Q1: 1 mm). A total of 187 (70%) aneurysms were bifurcation and 79 (30%) aneurysms were side walls. The mean packing density was 33.35% ± 0.38%. Angiographic parameters before endovascular coil embolization also have insignificant differences between the BSE cohort and LSE cohort (*p* > 0.05 for all, Fisher's exact test or Mann-Whitney test or unpaired *t*-test, Table 2).

**Table 1:** Demographical and clinical characteristics of patients and patients’ behavior before endovascular coil embolization.

**Parameters**		**Total**	**Cohorts**		**Comparisons between cohorts**	
			**BSE**	**LSE**		
**Stent-assisted embolization**		**Braided or Laser engraved**	**Braided**	**Laser engraved**		
Numbers of patients		266	125	141	**p-value**	**95% Cl**
Gender	Male	155 (58)	70 (56)	85 (60)	0.5338 (Fisher's exact test)	0.7058 to 1.177
	Female	111 (42)	55 (44)	56 (40)		
Age (years)		62 (67–57)	63 (67–58)	61 (67–55)	0.1115 (Mann-Whitney test)	N/A
Comorbidities						
Hypertension		119 (45)	57 (46)	62 (44)	0.8059 (Fisher's exact test)	0.8016 to 1.337
Diabetes		22 (8)	11 (9)	11 (8)	0.8257 (Fisher's exact test)	0.6900 to 1.660
Hyperlipidemia		80 (30)	35 (28)	45 (32)	0.5058 (Fisher's exact test)	0.6769 to 1.208
Patient's behavior						
Smoking	Current	19 (7)	11 (9)	8 (6)	0.3584 (*χ*^2^-test; df: 2)	N/A
	Previous	27 (10)	15 (12)	12 (9)		
	No smoker	220 (83)	99 (79)	121 (86)		

Variables were depicted as frequency (percentage) or median (Q3–Q1).

p less than 0.05 is considered significant.

CI, Confidence Interval (using the approximation of Katz.); N/A, Not Applicable; df, degree of freedom.

**Table 2:** Angiographic parameters before endovascular coil embolization.

**Parameters**		**Total**	**Cohorts**		**Comparisons between cohorts**	
			**BSE**	**LSE**		
**Stent-assisted embolization**		**Braided or laser engraved**	**Braided**	**Laser engraved**		
Numbers of patients		266	125	141	**p-value**	**95% CI**
Aneurysmal location	Anterior circulation	226 (85)	104(83)	122(87)	0.4942 (Fisher's exact test)	0.6321 to 1.215 (using the approximation of Katz.)
	Posterior circulation	40 (15)	21(17)	19(13)		
Aneurysm size (mm)		5.5 (7–4)	5 (7–3.5)	6 (7–4)	0.3159 (Mann-Whitney test)	N/A
Neck size (mm)		4 (5–3)	4 (5–3)	4 (5–3)	0.5666 (Mann-Whitney test)	N/A
Depth-to-neck (mm)		1.2 (1.3–1)	1.2(1.3–1)	1.2 (1.3–1)	0.46 (Mann-Whitney test)	N/A
Aneurysm type	Bifurcation	187 (70)	90 (72)	97 (69)	0.5929 (Fisher's exact test)	0.8140 to 1.450 (using the approximation of Katz.)
	Side-wall	79 (30)	35 (28)	44 (31)		
Packing density (%)		33.35±0.38	32.63±0.58	33.98±0.48	0.073 (unpaired *t*-test)	−0.1265 to 2.820 (df: 264)

Variables were depicted as frequency (percentage) or median (Q3–Q1) or median ± standard error of mean.

p less than 0.05 is considered significant.

CI, Confidence interval; N/A, Not Applicable; df, degree of freedom.

### Follow-up study

The follow-up period of patients was 37.71 ± 0.29 months, successful coil embolization procedure was 54%. The successful coil embolization procedure was 57% vs. 52% for the BSE and the LSE cohorts, respectively (*p* = 0.46, Fisher's exact test). The deployment success rate was 97%. The deployment success rate was higher in cases of laser engraved stent-assisted embolization than those of braided stent-assisted embolization (*p* = 0.0142, Fisher's exact test). There were no significant differences in follow-up time and results of procedure between patients in the BSE cohort and those of the LSE cohort (*p* > 0.05 for all, Fisher's exact test or unpaired *t*-test, Table 3).

**Table 3:** Follow-up study after endovascular coil embolization.

**Parameters**		**Total**	**Cohorts**		**Comparisons between cohorts**		
			**BSE**	**LSE**			
**Stent-assisted embolization**		**Braided or laser engraved**	**Braided**	**Laser engraved**			
Numbers of patients		266	125	141	**p-value**	**95% CI**	**df**
Follow-up period (months)		37.71 ± 0.29	37.69 ± 0.33	37.74 ± 0.44	0.9301 (unpaired *t*-test with Welch correction)	−1.062 to 1.161	247
Deployment success		257 (97)	117 (94)	140 (99)^a^	0.0142 (Fisher's exact test)	0.3922 to 0.6689 (using the approximation of Katz.)	N/A
Results of procedure	Successful procedure	144 (54)	71 (57)	73 (52)	0.46 (Fisher's exact test)	0.8597 to 1.443 (using the approximation of Katz.)	N/A
	Unsuccessful procedure	122 (46)	54 (43)	68 (48)			

Variables were depicted as frequency (percentage) or mean ± standard error of mean (SEM).

p less than 0.05 is considered significant.

CI, Confidence Interval; N/A, Not Applicable; df: degree of freedom.

The Raymond classification grading of complete occlusion and residual neck were considered a successful procedure.

a Significant higher.

### Adverse effects

A total of 4 (2%) patients have died during follow-up. Periprocedural intracranial hemorrhage was higher in patients who underwent braided-assisted embolization than those who underwent laser engraved assisted embolization (*p* = 0.0142, Fisher's exact test). Permanent morbidities were higher in patients who underwent laser engraved assisted embolization than those who underwent braided-assisted embolization (*p* = 0.0389, Fisher's exact test). The details of adverse effects are reported in Table 4. In-stent thrombosis was the common procedural-related adverse effect. A total of 7(3%) patients faced in-stent thrombosis. Among 7 patients, 4 (3%) were from the LSE cohort and 3 (2%) patients from the BSE cohort (*p* = 0.6683, Fisher's exact test, 0.6201 to 2.657: 95% CI).

**Table 4:** Disease-related adverse effects during follow-up study after endovascular coil embolization and periprocedural complication.

**Parameters**	**Total**	**Cohorts**		**Comparisons between cohorts**	
		**BSE**	**LSE**		
**Stent-assisted embolization**	**Braided or laser engraved**	**Braided**	**Laser engraved**		
Numbers of patients	266	125	141	**p-value**	**95% CI**
Periprocedural intracranial hemorrhage	9 (3)	8 (6)^a^	1 (1)	0.0142	1.495 to 2.550
Postprocedural intracranial hemorrhage	6 (3)	5 (4)	1 (1)	0.1023	1.233 to 2.644
Permanent morbidity	9 (3)	1 (1)	8 (6)^b^	0.0389	0.03611 to 1.468
Mortality	4 (2)	1 (1)	3 (2)	0.6249	0.09625 to 2.899

Variables were depicted as frequency (percentage).

Fisher's exact test was used for statistical analysis. p less than 0.05 is considered significant.

CI, Confidence Interval (using the approximation of Katz.).

a Braided stent-assisted adverse effect.

b Laser engraved stent-assisted adverse effect.

### Analysis according to aneurysmal location

Successful procedure for the BSE cohort was higher for posterior circulation aneurysmal location than those of the LSE cohort but it was not statistically significant (*p* = 0.7271, Fisher's exact test). A higher percentage of postprocedural intracranial hemorrhage and mortality was reported for the LSE cohort for posterior circulation aneurysmal location than those of the BSE cohort but these were not statistically significant (*p* > 0.05 for both, Fisher's exact test). The details of the analysis of procedural success rate and follow-up outcomes according to the aneurysmal location are reported in Table 5.

**Table 5:** Analysis of procedural success rate and follow-up outcomes according to aneurysmal location.

**Parameters**		**Cohorts**									
**Aneurysmal location**		**Anterior circulation**					**Posterior circulation**				
**Cohorts**		**Total**	**BSE**	**LSE**	**Comparisons between cohorts**		**Total**	**BSE**	**LSE**	**Comparisons between cohorts**	
Numbers of patients		226	104	122	**p**	**95% CI**	40	21	19	**p**	**95% CI**
Results of procedure	Successful procedure	115 (51)	55 (53)	60 (49)	0.5958	0.8161 to 1.438	29 (73)	16 (76)	13 (68)	0.7271	0.5873 to 2.508
	Unsuccessful procedure	111 (49)	49 (47)	62 (51)			11 (27)	5 (24)	6 (32)		
Postprocedural intracranial hemorrhage		4 (2)	5 (4)	0 (0)	0.02	1.920 to 2.572	1 (5)	0 (0)	1 (5)	0.475	Infinity to Infinity
Mortality		3 (2)	1 (1)	2 (2)	0.999	0.1447 to 3.599	1 (3)	0 (0)	1 (5)	0.475	Infinity to Infinity

Variables were depicted as frequency (percentage).

Fisher's exact test was used for statistical analysis. p less than 0.05 is considered significant.

CI, Confidence Interval (using the approximation of Katz.).

#### Recanalization

A total of 64 (51%) and 58 (59%) patients from the BSE and the LSE cohorts underwent recanalization. There were no significant differences in the rate of recanalization between both cohorts (0.2928, χ^2^-test, Table 6).

**Table 6:** Recanalization during follow-up study after endovascular coil embolization.

**Parameters**		**Total**	**Cohorts**		**Comparisons between cohorts**	
			**BSE**	**LSE**		
**Stent-assisted embolization**		**Braided or laser engraved**	**Braided**	**Laser engraved**		
Numbers of patients		266	125	141	**p-value**	**df**
Recanalization	Within 6 months	60 (23)	27 (22)	23 (16)	0.2928 (χ^2^-test)	3
	6 to 12 months	41 (15)	21 (17)	20 (14)		
	12 to 18 months	33 (12)	18 (14)	15 (11)		
	Not recanalized	132 (50)	64 (51)	83 (59)		
Numbers of recanalization		1 (1–1)	1 (1–1)	1 (1–1)	0.4576 (Wilcoxon matched-pairs signed-ranks test)	N/A

Variables were depicted as frequency (percentage) or median (Q3–Q1).

p less than 0.05 is considered significant.

CI, Confidence Interval; N/A, Not Applicable; df, degree of freedom.

## Discussion

After approval from the United State Food and Drug Administration (USFDA), varieties of stents were available for coil embolization purposes. There are different classifications of stents, for example, cell type, open or closed type, manufacturing method, braided or laser-cut type, metal coverage type, cell size type, visibility type, and delivery system type. The stenting system is continuously evolving for tailor-made coil embolization and successful deployment.^10^ Stent which has higher deployment success even with higher procedural complication was preferred most.^12^ Proper selection of stent during coil embolization procedure increases deployment success and decreases procedural complications and adverse effects.

The current study found that laser engraved stent-assisted embolization had a high deployment success rate and fewer periprocedural intracranial hemorrhage rates than braided stent-assisted embolization. Deployment and the follow-up results of the current study are in line with the results of the retrospective review study,^14^ multi-center analysis,^15^ and comparison studies.^10^,^13^,^17^,^18^ Laser engraved stent has technical feasibility in applying stent-through compared to a braided stent in the procedure of stent-assisted coil embolization.^12^ Higher metal surface coverage of laser engraved stent increases flow diversion that decreases adverse effects^13^ Laser engraved stent has fewer problems with deployment and may have better follow-up outcomes after embolization.

A higher% of patients from the LSE cohort were recanalized than those of the BSE cohort. The results of the recanalization of the current study were not consistent with those of a retrospective study^19^ and a comparative study.^10^ Laser engraved stent-assisted embolization procedures requires a recanalization procedure than braided stent-assisted embolization procedures. This would be a topic for further research.

The coil embolization procedure (whole endovascular coil embolization procedure) success rate was fewer for laser engraved stent-assisted embolization than braided stent-assisted embolization but statistically insignificant between cohorts. The results of the coil embolization procedure success rate of the current study were consistent with those of a comparative study^10^ and a case series.^20^ Laser engraved stent offers ∼23% of metal coverage and braided stent offers ∼10% of metal coverage.^21^ The porosity of a neurovascular stent controls circulatory hemodynamics.^22^,^23^ Laser-engraved stent-assisted embolization has incomplete expansion in tortuous arteries compared to braided stent-assisted embolization.^12^ The success of coil embolism would be higher for braided stent-assisted embolization than the laser-engraved stent-assisted embolization.

In posterior circulation, aneurysmal location braided stent-assisted embolization had a higher success rate and higher favorable outcomes compared to laser engraved stent-assisted embolization. The results of the outcome according to the aneurysmal location of the current study were in line with those of comparative studies.^10^,^24^ A braided stent provides a moderate flow-diversion effect, that is advantageous for posterior circulation aneurysmal embolization.^24^ The laser engraved stent-assisted coil embolization is technically easier to use with improved outcomes at follow-up, braided stent-assisted embolization is more effective for cerebral artery aneurysms of the posterior circulation.

Permanent morbidity was significantly fewer in patients who underwent braided stent-assisted embolization than those who underwent laser engraved stent-assisted embolization. The results of the permanent morbidity of the current study were in line with those of a comparative study.^13^ Demographical and clinical characteristics of patients, patients’ behavior, angiographical characteristics, coil embolization procedure, and follow-up parameters may affect morbidity after embolization. However, further research is required for reasons of permanent morbidity after embolization.

The limitations of the study are a retrospective analysis and a lack of dynamic study. The stent morphological characters did not consider. The recanalization rate was 50% which was overstating the situation than those in available studies.^13^,^25^

## Conclusions

This observational study compared the braided stents and the laser-cut stents assisted coil embolization among Chinese patients with complex intracranial aneurysms. This study supports recent findings from the literature that braided stent-assisted coil embolization and laser engraved stent-assisted coil embolization was safe and effective in treating unruptured intracranial aneurysms. The study found that the laser-cut stents-assisted coil embolization showed a higher deployment success rate, as well as a much lower rate of periprocedural intracranial hemorrhage. Unfortunately, laser-cut stents-assisted coil embolization could increase the chance of permanent morbidities. The advantages of braided stents-assisted coil embolization include a higher success rate, a lower risk of postprocedural intracranial hemorrhage, and mortality. The study evaluated the pros and cons of both methods and provided valuable practical clinical suggestions to optimize the application of both methods to the most fitful cases but avoid potential risks.

## Availability of data and materials

The datasets were used and analyzed during the current study available from the corresponding author on reasonable request.

## Authors’ contributions

All authors have read and approved the manuscript for publication. JZ contributed to visualization, validation, methodology, and project administartion of the study. LH contributed to methodology, resources, conceptualization and software of the study. LZ contributed to investigation, supervision, validation, methodology, and formal analysis of the study. KY contributed to formal analysis, data curation, validation, and methodology of the study. XX contributed to methodology, software, and visualization of the study, and original drafted, review, and edited the manuscript for intellectual content. All authors agree to be accountable for all aspects of work ensuring integrity and accuracy.

## Funding support

This study was supported by the Research and Application of an AI-based Intelligent Assistance System for Intracranial Aneurysm Clipping Surgery (No: 2021YFG0316).

## Conflicts of interest

The authors declare no conflicts of interest.
